# Coordinated autophagy modulation overcomes glioblastoma chemoresistance through disruption of mitochondrial bioenergetics

**DOI:** 10.1038/s41598-018-28590-9

**Published:** 2018-07-09

**Authors:** Jurgen Kriel, Kristian Müller-Nedebock, Gerald Maarman, Siyasanga Mbizana, Edward Ojuka, Bert Klumperman, Ben Loos

**Affiliations:** 1 Department of Physiological Sciences, Faculty of Science, University of Stellenbosch, Stellenbosch, 7600 South Africa; 2 Department of Physics, Faculty of Science, University of Stellenbosch, Stellenbosch, 7600 South Africa; 3 Division of Exercise Science and Sports Medicine Institute (ESSM), Department of Human Biology, University of Cape Town, Cape Town, 7700 South Africa; 4 Department of Polymer Science, Faculty of Science, University of Stellenbosch, Stellenbosch, 7600 South Africa

## Abstract

Glioblastoma Multiforme (GBM) is known to be one of the most malignant and aggressive forms of brain cancer due to its resistance to chemotherapy. Recently, GBM was found to not only utilise both oxidative phosphorylation (OXPHOS) and aerobic glycolysis, but also depend on the bulk protein degradation system known as macroautophagy to uphold proliferation. Although autophagy modulators hold great potential as adjuvants to chemotherapy, the degree of upregulation or inhibition necessary to achieve cell death sensitisation remains unknown. Therefore, this study aimed to determine the degree of autophagy modulation necessary to impair mitochondrial bioenergetics to the extent of promoting cell death onset. It was shown that coordinated upregulation of autophagy followed by its inhibition prior to chemotherapy decreased electron transfer system (ETS) and oxidative phosphorylation (OXPHOS) capacity, impaired mitochondrial fission and fusion dynamics and enhanced apoptotic cell death onset in terms of cleaved caspase 3 and cleaved PARP expression. Therefore, coordinated autophagy modulation may present a favourable avenue for improved chemotherapeutic intervention in the future.

## Introduction

Globally, Glioblastoma Multiforme (GBM) presents as both the most prevalent and invasive form of Central Nervous System (CNS) malignancy. Patient life expectancy has remained largely unchanged over the past three decades, with a mean survival time of only 15 months^1^. This has been attributed to the rapid tumour recurrence and resistance to cell death after exposure to chemotherapy, radiation and surgical removal. Initial attempts to identify the key genetic markers associated with resistance led to the identification of enhanced DNA repair through MGMT mediated signalling in highly malignant tumours^2^. Cell cycle and angiogenesis related molecular regulators such as AKT, PTEN and Ras have also shown to be frequently mutated in these tumours^3^. However, combining growth factor receptor inhibitors or anti-angiogenic reagents with chemotherapy has not been able to enhance mean patient survival time^4^. Furthermore, excessive exposure to chemotherapy and radiation has been shown to decrease patient quality of life following treatment, contributing to decreased patient survival time^4^.

This has led to a resurgence in studies focussing on the metabolic upkeep of GBM pathogenesis and resistance^5^. The involvement of macro-autophagy (hereafter referred to as autophagy) in upholding healthy cell metabolism under nutrient limiting conditions has garnered much interest with regards to its role in tumour bioenergetics^6^. Mammalian target of rapamycin (MTOR) dependent induction of autophagy results in the bulk degradation of long lived or damaged cytosolic proteins and organelles. This provides key metabolic substrates for glycolysis and the tricarboxylic acid (TCA) cycle, thereby making it an excellent energy reservoir to uphold tumour proliferation under hypoxic or cytotoxic conditions^7^. In this regard, autophagy induction has been observed in response to treatment of glioma cells with the standard of care chemotherapeutic Temozolomide (TMZ)^8^. However, given the molecular crosstalk between regulators of apoptosis and autophagy, enhanced GBM cell death onset has been observed in recent studies combining either autophagy inducers (such as Rapamycin or Temsirilomus) or inhibitors (such as Hydroxychloroquine or Bafilomycin) with chemotherapy^9,10^. Furthermore, current phase 1 clinical trials focussing on the adjuvant effects of such modulators in chemotherapy pay little attention to the involvement of autophagy in key metabolic pathways.

Current evidence suggests that both oxidative and glycolytic metabolic pathways are involved in glioma progression, depending on their level of malignancy^11–13^. In the context of chemotherapeutic resistance, glioma cells have been shown to depend on enhanced electron transport system (ETS) coupling and autophagy to acquire resistance to TMZ^10,14–16^. The mitochondrial network operates as a highly energetic reticulum subjected to continuous and rapid remodelling through fission and fusion events. Although evidence exists for the involvement of the fission and fusion machinery in metabolic sensing and ETC efficiency, their role in tumour metabolism remains unclear^17,18^. Therefore, this study aimed to: (i) determine the degree of autophagy modulation necessary to sensitise glioma cells to chemotherapy; (ii) assess mitochondrial bioenergetics in terms of topology, fission and fusion dynamics and electron transport system efficiency; (iii) assess whether changes in autophagic flux results in an altered mitochondrial bioenergetic phenotype and (iv) determine the extent of diminished mitochondrial bioenergetic capacity necessary to achieve cell death sensitisation.

## Materials and Methods

### Cell Culture

U-118MG and U-87 cells were purchased from the American Type Culture Collection (ATCC) and supplemented with Dulbecco’s Modified Eagles Medium (DMEM), 1% penicillin/streptomycin (PenStrep) (Life Technologies, 41965062 and 15140122) and 10% foetal bovine serum (FBS) (Scientific Group, BC/50615-HI) and incubated in a humidified incubator (SL SHEL LAB CO_2_ Humidified Incubator) in the presence of 5% CO2 at 37 °C. 3D spheroids were generated by coating 96 well plates with 50 µl of 0.1% agarose solution per well, leaving the agarose to solidify under UV light 1 hour prior to seeding (2 × 10^3^ cells per well). Spheroids were incubated for a maximum of 72 hours prior to treatment.

### Reagents

The autophagy modulating drugs, Hydroxychloroquine Sulfate (HCQ) and Rapamycin, as well as the chemotherapeutic Temozolomide (TMZ) were purchased from Sigma-Aldrich (1327000, R8781 and T2577). Bafilomycin A_1_ was acquired from LKT laboratories (B0025). HCQ and Bafilomycin A_1_ were dissolved in H_2_0, whist Rapamycin and TMZ were prepared in dimethyl sulfoxide (DMSO) (Sigma-Aldrich, D2650). Primary antibodies for LC3, cleaved-Caspase3, cleaved-PARP and β-Actin were obtained from Cell Signalling (2775, 9541 S, 56416, 4970). ATG5 primary antibody was obtained from Santa Cruz (sc-8666) and TOMM-20 from abcam (ab56783). The mitochondrial primary antibodies OPA-1, Drp1, MFN-1, MFN-2 and secondary antibodies (anti-rabbit, anti-mouse and anti-goat) were purchased from Abcam (ab157457, ab56788, ab57602, ab56889, ab78547, ab97110). All compounds for the mitochondrial respiration medium (MiRO5) were purchased from Sigma-Aldrich unless otherwise specified. MiRO5 medium consisted of EGTA (E4378), MgCl_2_ (M8266), Lactobionic acid (153516), Taurine (T0625), KH_2_P0_4_ (Merck, 104873), HEPES (H7523), D-Sucrose (84097) and BSA (10735078001). The substrates, uncouplers and inhibitors used during the SUIT protocol were supplied by Sigma-Aldrich, including L-Glutamic acid (G1626), L-Malic acid (M1000), Pyruvic acid (P2256), Succinate (S2378), Ascorbate (A4034), Tetramethyl-p-phenylenediamine dihydrochloride (TMPD, T3134), Adenosine 5’diphosphate (ADP, A2754), Carbonyl cyanide m-chloro phenyl hydrazine (CCCP, C2759) and Antimycin A (A8674). Cell permeabilization was achieved through titration of Digitonin (D141, 10 mg dissolved in 1 mL DMSO).

### Synthesized Polymer

Nanocarriers were prepared via self-assembly of block copolymers consisting of polyethylene glycol (PEG) as the hydrophilic block and the polypeptide as hydrophobic block. The self-assembly into micellar aggregates typically results from interactions with the bioactive molecules or induced by a pH change^19–21^. Bioactive molecules are normally bound to or adsorbed in the core through physical interactions such as hydrophobic interactions or electrostatic interactions^22,23^. A novel nano carrier was prepared by covalently attaching hydroxychloroquine via an acid-labile β-thiopropionate bond to poly(N-vinylpyrrolidone-block-(cysteine-co-glycine)).

### Cell Viability Assays

Mitochondrial reductive capacity was used as an indicator for cell viability, and measured using water-soluble tetrazolium bromide (WST-1) (Roche, 11644807001). Cells were seeded in 48-well plates, where after cell culture media containing treatment reagents was aspirated and replaced with 200uL of fresh media. 5 uL of WST-1 was added to each well and incubated at 37 °C for 80 minutes, after which the 48-well plate was placed in a multiplate reader (EL-800, Bio-Tek instruments Inc.) and absorbance values read at 480 nm. Reductive capacity was calculated in percentage relative to the untreated control group. Apoptosis was further quantified by propidium iodide (PI) exclusion assays using flow cytometry (BD FACS Aria). Prior to cell sorting, treated cells were trypsinized, centrifuged (1100 rpm, 3 min) and resuspended in PI staining solution (2ug/mL diluted in PBS) for 15 minutes. Analysis was conducted using FACS Diva software. Spheroids were stained with PI for 1 hour prior to imaging and viability was measured as the mean fluorescence intensity using ImageJ (version 1.51, National Institutes of Health, USA).

### Transfection

Cells were transfected with mitochondrial associated photoactivatable green fluorescent protein (mito-PA-GFP) or ATG siRNA (Hs_APG5L_5 FlexiTube siRNA, Qiagen, NM_004849) through electroporation utilizing the Neon^®^ Transfection System (Life Technologies). Transient transfection was achieved as per the manufacturers guidelines at a final voltage of 1300 V, 30 ms pulse width and cell density of 5 × 10^5^.

### Confocal Microscopy

#### Photoactivation Assay

In order to determine the rate at which mitochondrial fission and fusion occurs, the spread of mito-PA-GFP from a subset of mitochondria throughout the mitochondrial network was tracked over time, as previously described by Karbowski *et al*. (2004), using a Carl Zeiss Confocal Elyra PS1 microscope with LSM 780 technology^24^. Transfection with mito-PA-GFP and staining with tetramethylrhodamine-ethyl ester (TMRE) (Life Technologies, T669) allowed for visualisation of the mitochondrial network. Following transfection, cells were treated accordingly and stained with 100 nM TMRE. For each cell, 2–3 regions of interest (ROI) of the cross-section area in a single focal plane was selected for photoactivation. Once selected, these regions were exposed to 403 nm laser stimulation at 100% intensity, resulting in the activation of mito-PA-GFP observed at 60× magnification. Only regions that displayed an increase in signal intensity of at least twice that of the initial intensity were tracked over time. Mito-PA-GFP signal distribution was observed under 488 nm excitation and a live cell time lapse was constructed with images acquired every second for 300 cycles (10 minutes) using an iteration speed of 15. Image processing was conducted with ZEN software (black edition, 2011, version 7.04.287). A detailed example of signal decay resulting from individual fission and fusion events is illustrated in Supplementary Fig. 1.

#### Morphometrics

To assess the morphometric characteristics of mitochondrial networks, live cell confocal microscopy was conducted with z-stacks acquired every 30 seconds for 10 minutes (Carl Zeiss LSM 780) in order to produce mean intensity projections for image analysis. Prior to imaging, media was removed followed by treatment with 200 μL TMRE working solution. Image processing was conducted in Wolfram Mathematica (Version 10.2) to generate graph-like binarized micrographs. These were used to assess the degree of connectivity within the mitochondrial networks (calculated as the total number of vertices by the total number of overlapping vertices within a graph like structure) and to determine the degree of scaling (given by a linear gradient fitted to a log[Area] log[Perimeter] scatter plot. Spheroid size determination was conducted prior to live cell imaging (Olympus IX81 widefield microscope) with ImageJ (version 1.51, National Institutes of Health, USA).

### Western Blot Analysis

Total cell protein was extracted utilising a modified radio immunoprecipitation (RIPA) buffer consisting of 20 mM Tris-HCl (pH 7.4), 137 mM NaCl, 10% Nonidet-P40 and 10% Na-deoxycholate, 42 µL complete EDTA-free protease inhibitor tablet solution, 1 mM PMSF, 1 mM Na_3_VO_4_ and 1 mM NaF phosphatase inhibitors. Protein concentration was determined using a Bradford Assay^25^. Cell lysates were diluted in Laemmeli sample buffer, boiled for 5 min and 50 µg protein was separated by 12% SDS-PAGE-gel electrophoresis (Bio Rad Mini-Protean ®TGX™ fast cast system). Proteins were transferred onto a PVDF (poly vinydilene difluoride) membrane using BIO-RAD Trans-Blot transfer packs and a BIO-RAD Trans-Blot turbo transfer system. Membranes were blocked for 60 minutes in 5% non-fat milk made up in 1 × TBS-T (Tris-buffered saline and 1% Tween20), followed by incubation with appropriate primary and secondary anti-bodies. Band intensities were detected with a BIO-RAD Chemidoc MP imaging system using Image Lab software (version 4.1) and expressed as a percentage relative to band intensities of untreated control cells. β-actin was used as a loading control for all membranes.

### Bafilomycin A1 Treatment for Western Blot Analysis of LC3

Bafilomycin A1 impairs the fusion of lysosomes with autophagosomes by inhibiting H^+^ATP-ase activity. A 100 µM stock solution was prepared in DMSO and diluted to 400 nM in normal growth media. Cells were seeded in 25 cm^2^ flasks at a density of 10 × 10^6^ cells and treated accordingly, followed by incubation with 400 nM Bafilomycin 4 hours prior to protein extraction. RIPA protein extraction, Braford analysis, sample preparation and Western Blot analysis of LC3 and β-Actin was performed as described above.

### High resolution respirometry

Measurement of mitochondrial respiration was performed with a polarographic oxygen sensor in 2 ml glass chambers of an Oxygraph 2 K (Oroboros Instruments, Innsbruck, Austria). The amplified signal from the oxygen sensor was recorded on a computer at sampling intervals of 2 sec using DatLab acquisition software (Oroboros Instruments, Innsbruck, Austria). Before all experiments were commenced, calibration of the respirometer was performed at air saturation, 37 °C. The electron transfer system (ETS) and oxidative phosphorylation (OXPHOS) capacities were determined through stepwise titration of specific substrates and inhibitors (SUIT protocol). In accordance with the Oroboros guidelines (Gnaiger, 2014), the resulting O_2_ flux values were utilized to calculate routine respiration, the non-phosphorylating resting state (LEAK), OXPHOS capacity through complex 1, residual oxygen consumption (ROX) and complex IV activity for normalisation. Following permeabilization with 1.5 mM Digitonin, Pyruvate (5 mM), Malate (2 mM) and Glutamate (10 mM) were added to determine the LEAK state. Complex I linked OXPHOS was measured by addition of 2.5 mM ADP. ETS capacity was determined through subsequent titrations of 0.5 µM CCCP, until a maximal oxygen flux was reached, which was taken as the maximal ETS capacity. Correcting for ROX, involved the addition of complex III inhibitor Antimycin-A and the resulting flux was subtracted from all final values. Complex IV activity was used as a surrogate marker for mitochondrial number, and therefore subtracted from all the final ETS and OXPHOS capacities^26^. To achieve this, titration of TMPD (0.5 mM) and ascorbate (2 mM) to assess complex-IV-linked respiration was performed as a proxy for mitochondrial content. Oxygen flux at all respiratory states was normalized to the complex-IV flux to correct for variations in cell content in the oxygraph chambers.

### Lactate Determination

U-118MG cells were seeded in 6- well plates at a density of 10 × x 105 cells per well. After treatment, 2 mL of culture medium was used for lactate determination. Lactate determination required addition of 5 µl sample or lactate standard (0–5 mM) to 95 µl PBS (1 mM), NAD + (4 mM), LDH (4 U/mL) and hydrazine (320 mM) in a 96-well plate, incubated for 90 minutes at 37 °C. Absorbance values were read at 340 nm using a BioTek Powerwave 340 spectrophotometer.

### Statistical Analysis

All statistics were conducted in Statistica (13.0) utilising a one-way ANOVA together with Bonferonni and LSD Post-Hoc Tests where p < 0.05 was considered significant.

### Data availability

The datasets analysed in the current study are available from the corresponding author on reasonable request.

### Decreased cell viability is associated with decreased autophagic flux

**Figure 1:**
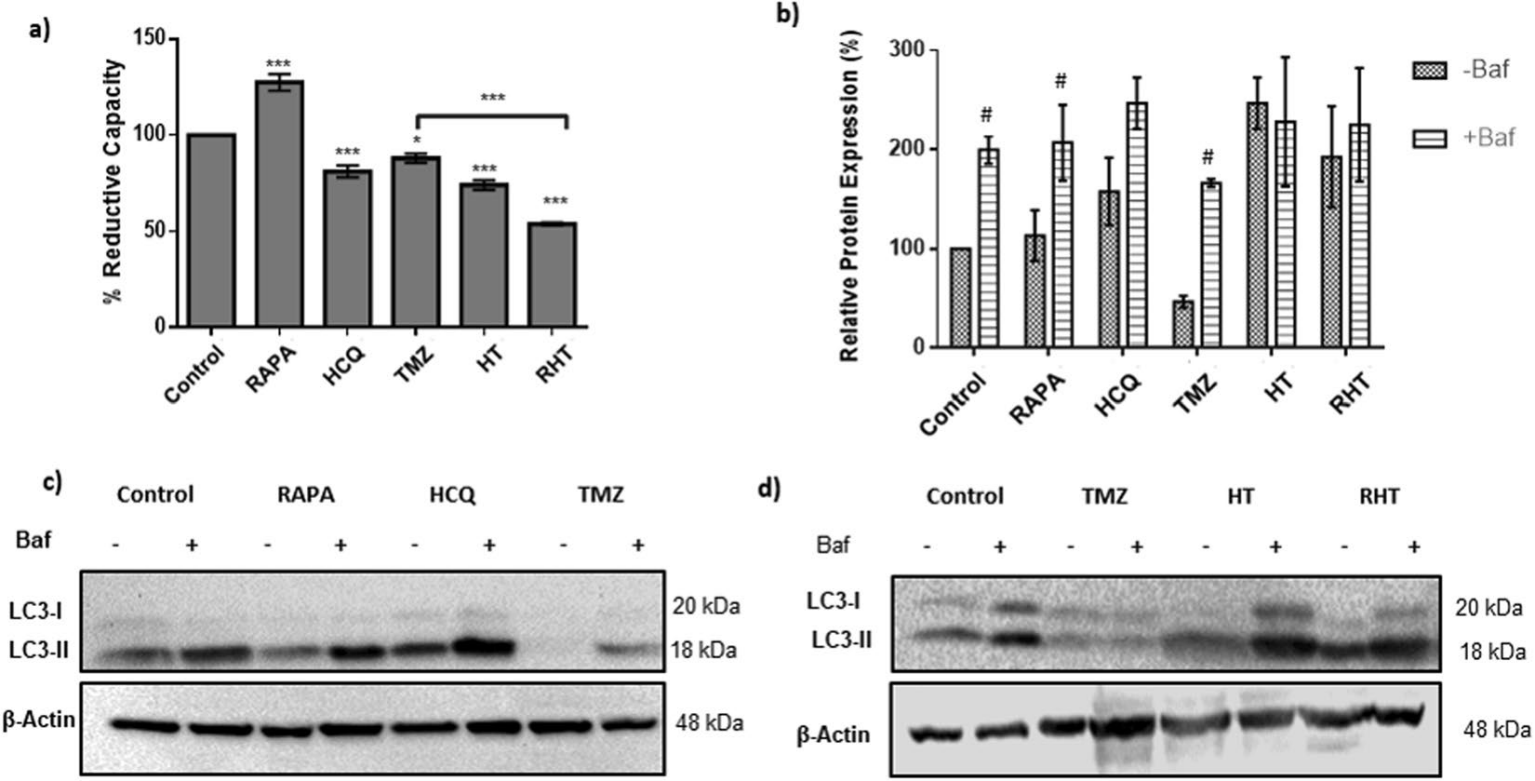
Decreased cell viability is associated with decreased autophagic flux. (**a**) WST1 viability assay, (**b**) Relative protein expression levels of LC3 and corresponding representative immunoblots in the presence and absence of Bafilomycin (Baf) treatment (400 nM, 2 hours) for (**c**) Control, 50 nM Rapamycin (Rapa), 50 µM Hydroxychloroquine (HCQ) and 250 µM Temozolomide (TMZ) and (**d**) Control, 250 µM TMZ, treatment HCQ (50 µM) + TMZ (250 µM) (HT) and Rapa (50 nM) + HCQ (50 µM) + TMZ (250 µM) (RHT) treatment groups. All error bars, ±SEM. ***p < 0.001, *p < 0.05 vs Control n = 6, ^#^p < 0.05 vs corresponding Baf negative group n = 3.

WST-1 viability assays were conducted to assess the effects of autophagy modulators (HCQ and Rapamycin) as well as TMZ on cell viability, represented by the percentage mitochondrial reductive capacity. Incubation of glioma cells for with the autophagy inducer Rapamcyin (50 nM, 6 hours) led to a significant increase in reductive capacity (Fig. 1a). Conversely, treatment with HCQ (50 µM) for the same duration decreased cell viability (Fig. 1a) and incubation for 24 hours with TMZ (250 µM) led to a moderate decrease in reductive capacity (Fig. 1a) when compared to the untreated control. This indicates that although the U-118MG cell line was sensitive to chemotherapy, failure to reach more than a 50% decrease in reductive capacity after prolonged incubation made it possible to investigate the sensitisation effects of adjuvant treatments. Two adjuvant treatment groups were investigated. The first was incubation of 50 µM HCQ for 6 hours followed by 250 µM TMZ for 24 hours (HT). The second group consisted of 6 hours Rapamycin pre-treatment, followed by incubation of 50 µM HCQ and 250 µM TMZ for and 24 hours respectively (RHT) (Fig. 1a).

HT treatment decreased reductive capacity significantly when compared to the control, although no significant difference was observed in comparison to TMZ treatment (Fig. 1a). However, dual modulation through RHT treatment proved to have the most prominent effect, with a substantial decrease in reductive capacity observed when compared to both individual modulation groups and the TMZ group (Fig. 1a). Of note, dual modulation led to a decrease in the percentage reductive capacity, which was of the same level as that of cells treated for 48 hours with TMZ (500 µM) (Supplementary Fig. 2). Furthermore, this sensitization effect was achieved through the use of Rapamycin and HCQ concentrations which were not inherently highly toxic to glioma cells *in vitro*. Seeing as many clinical trials currently focus on the adjuvant use of HCQ with chemotherapy, the HCQ adjuvant group was selected in addition to the dual modulation group for further analyses.

Inhibition of lysosomal fusion through BafilomycinA_1_ (Baf) treatment results in the accumulation of autophagosomal vacuoles, which can be measured by quantifying the relative levels of LC3-II protein present in the cell lysate. Quantifying the change in LC3-II in the presence and absence of Baf (400 nM) indicates the relative amount of autophagosome production present in a population of cells, serving as an indicator of the rate of protein degradation through autophagy i.e. autophagic flux. Significantly increased LC3-II protein levels were observed for the Baf positive Control, Rapamycin and TMZ groups when compared to their corresponding Baf negative expression levels, representative of an increased autophagic flux following Rapamycin and TMZ treatment (Fig. 1b–d). No significant increase was observed in the presence of Baf for the HCQ, HT and RHT groups when compared to their Baf untreated LC3-II expression levels, which suggests that autophagosome degradation was impaired (Fig. 1b–d).

Given that LC3-II is a reliable marker for autophagosome production, our data shows that an accumulation of LC3-II (decreased autophagic flux) is associated with diminished cell viability.

### Autophagy modulation, chemotherapy and its combination disrupts mitochondrial fission and fusion dynamics

**Figure 2:**
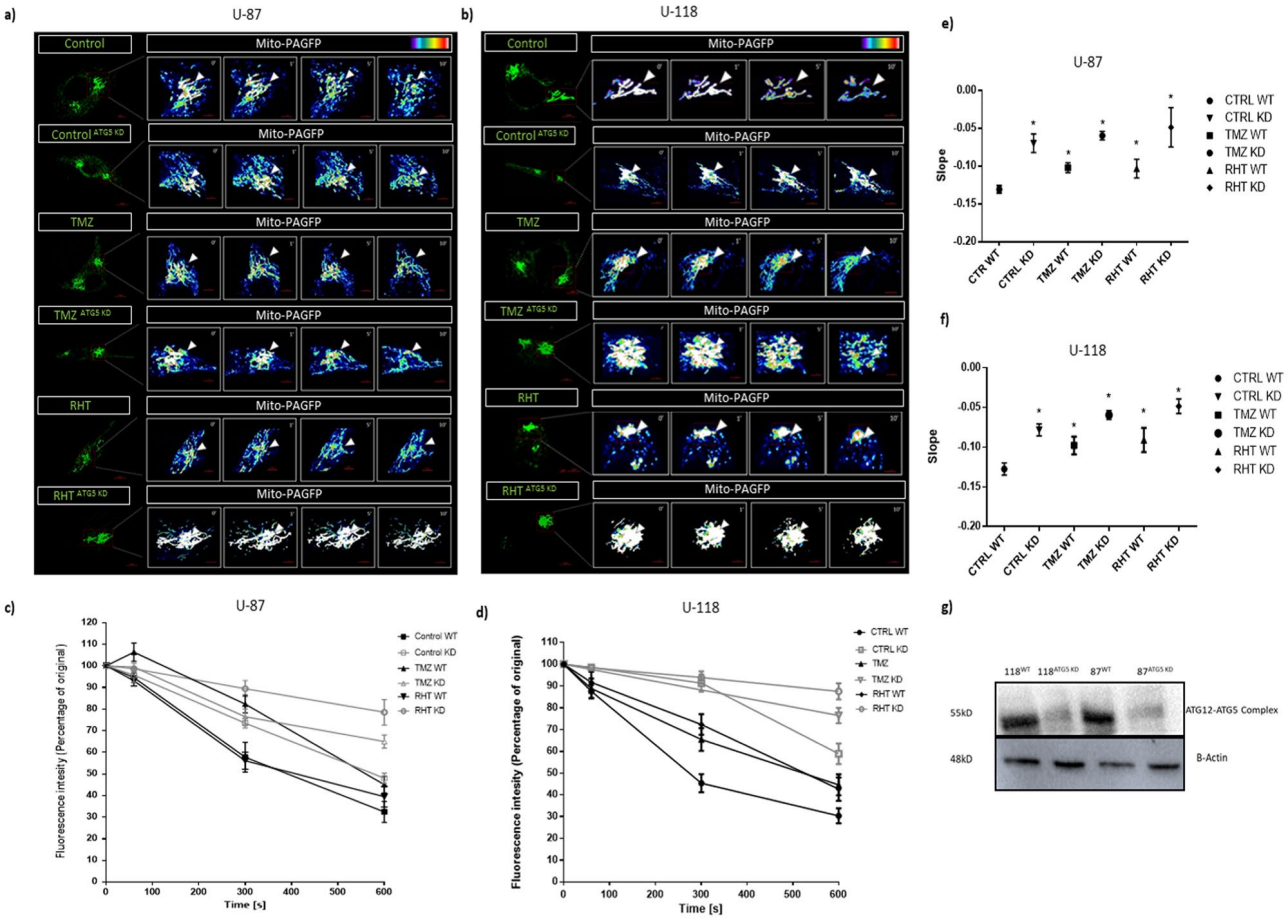
Autophagy modulation impairs mitochondrial dynamics. Representative images of mito-PA-GFP transfected cells (60× magnification, scalebar 5 μM) with the activated region outlined in red enhanced to display lookup table (LUT, linear range) intensities at 0, 1, 5 and 10 minute intervals post activation for (**a**) U-87 cells and (**b**) U-118 MG cells treated with TMZ, HT and RHT for both wild-type (WT) and ATG5 knockdown (KD) conditions. Mito-PA-GFP signal decay over time is represented as a percentage of the initial signal intensity in (**c**) and (**d**) for each cell line with corresponding slope comparisons of linear regressions through signal decay curves shown in (**e**) and (**f**). N = 6 *p < 0.05 vs control. (**g**) Representative immunoblot conducted to confirm ATG5 knockdown.

Next, we assessed whether changes in autophagic flux is associated with an altered mitochondrial phenotype. The potential of mitochondrial subunits to fuse with one another has been shown to correlate with healthy mitochondrial functionality. Therefore, we quantified the rate at which mitochondrial mitochondrial fission and fusion occurs for each treatment group, given by the decay in signal intensity of activated mito-PA-GFP over time. Fluorescence intensity was measured every second for 600 seconds and plotted over time as the percentage of initial signal intensity post activation (Fig. 2).

Linear regression through signal decay curves indicated that the fastest signal dissipation rate was displayed by Control cells and those treated with Rapamycin, indicating functional fission and fusion as the percentage fluorescence intensity reached below 50% after 300 seconds (Fig. 2a–c). However, signal dissipation rate was significantly impaired in the TMZ and RHT groups with a 50% decrease only observed after the full and 600 second time lapse suggesting that mitochondrial fission and fusion was impaired (Fig. 2b,c). Of note, although a large degree of fragmentation was observed in the HCQ, HT and RHT groups, the same decay curve was obtained for the intermediately fragmented network of the TMZ group (Fig. 2, Supplementary Fig. 2). Impaired fusion capability was therefore shown for treatment groups that displayed both impaired (HCQ, HT and RHT) and enhanced (TMZ) autophagic degradation. To rule out cell line specificity, TMZ and RHT treatments were carried out on U-87 cells, which also presented with slower mito-PA-GFP signal dissipation rate in response to these treatments (Fig. 2e).

To ascertain whether these changes can be attributed to autophagic signalling, photoactivation was also performed on ATG5 siRNA knockdown cells. Conflicting evidence exists for the effect of ATG5 knockdowns on mitochondrial network morphology, as decreased and increased tubulation has been reported in separate studies^27,28^. However, ATG5 has been implicated in mitochondrial quality control possibly through regulating mitophagy^29^. Therefore, even though structural changes might not be apparent, it is possible that functional responses in knockdown cells will be different. Indeed, our data indicate that the signal dissipation rate of activated mito-PA-GFP is slower in ATG5 siRNA knockdown U-87 and U-118 cells than in their wild type counterparts (Fig. 2,e,f). Furthermore, the fision and fusion response to both TMZ and RHT treatment was also altered, with a further decrease in signal dissipation rate observed for TMZ and RHT treated ATG5 knockdown cells that in their wild-type counterparts. These data indicate that complete inhibition of autophagy results in abrupt changes in mitochondrial dynamics and that the altered signal dissipation rate observed in the TMZ and RHT groups can be attributed to changes in autophagic flux.

### Coordinated autophagy modulation impairs mitochondrial network connectivity

**Figure 3:**
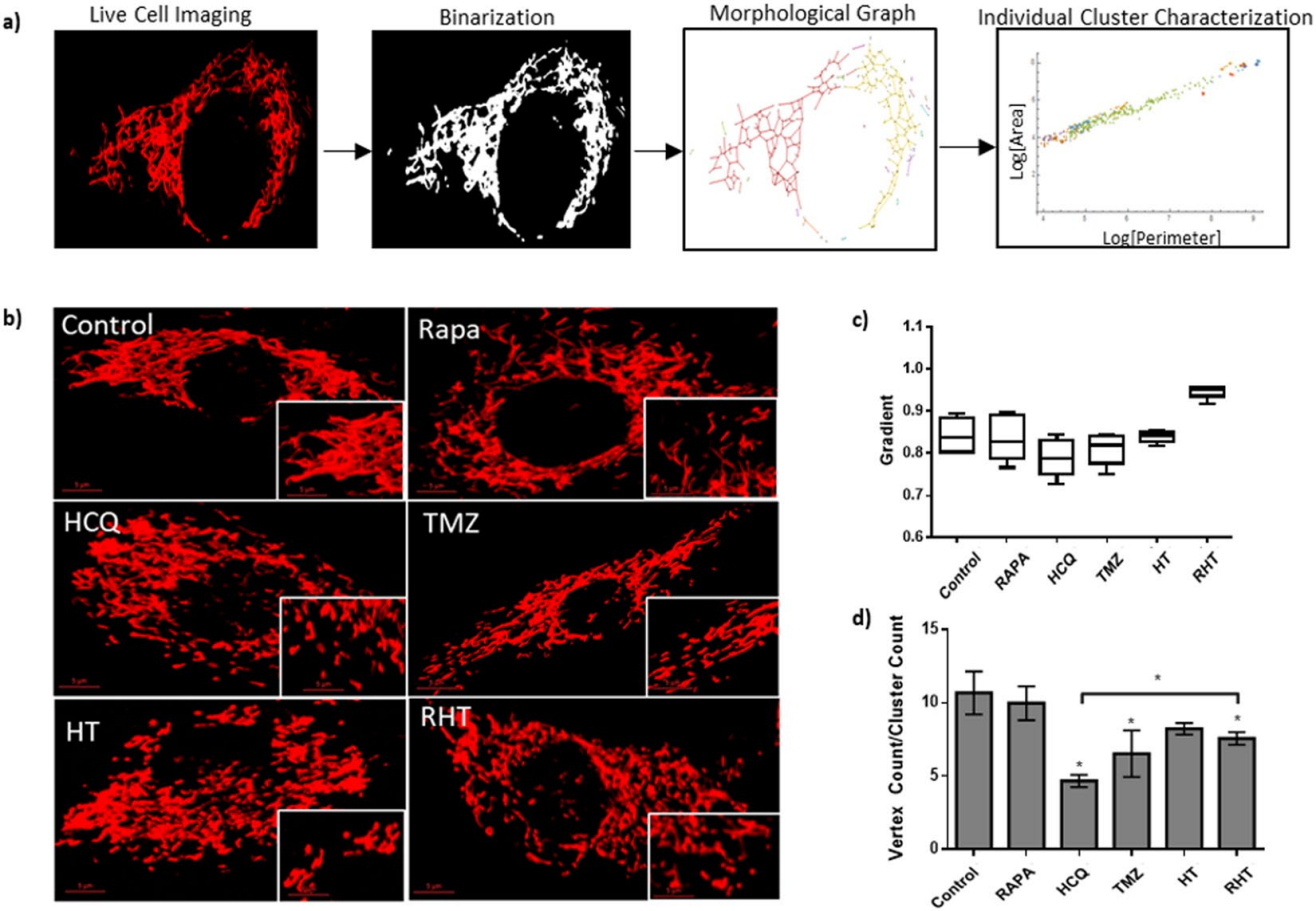
Coordinated autophagy modulation impairs mitochondrial network connectivity. (**a**) Maximal intensity projections of glioma cells stained with TMRE (red) at 60× magnification (scale bar 5 μM), (**b**) Mean gradients of power law curve fittings and (**c**) Connectivity Index (Vertex Count/Cluster Count, CI) values for Control (C), Rapamycin (R), Hydroxychloroquine (HCQ), Temozolomide (TMZ), HCQ and TMZ in combination (HT) and Rapamycin, HCQ and TMZ in combination (RHT) treatment groups. N = 6, *p < 0.05.

In order to relate mitochondrial network dynamics to their topology, the connectivity between mitochondrial subunits was quantified in terms of the degree of scaling within these networks and the amount of overlap between membrane structures. Scaling invariance was calculated by fitting a power law relationship to scatter plots of the outer perimeter and inner area of each network cluster (Fig. 3). With regards to the pure network properties of the mitochondrial networks the ratio of the total number of vertices to the number of connected clusters for a given graph was assessed, providing a “connectivity index” (Fig. 3).

The mitochondrial networks of U-118MG cells under control conditions were found to be highly connected (Fig. 3). Less individual clusters were found for the Rapamycin treatment group, although the overall connectivity was unaltered (Fig. 3). HCQ treatment resulted in a significant increase in fragmentation, with a large number of unconnected clusters (Fig. 3). Significantly more clusters were also observed in cells treated with, which were considerably less connected than the control. HCQ pre-treatment followed by incubation with TMZ led to a decreased cluster count, with networks being significantly more connected than the TMZ treatment group, but less than the control (Fig. 3). Rapamycin pre-treatment followed by HT decreased the number of mitochondrial network clusters significantly, although they displayed the same intermediate degree of connectivity as that of the HT group.

Comparing the degree of scale invariance, a power law relationship was present for all treatment groups in terms of their area and perimeter measurements (Fig. 3c). Importantly, the RHT group yielded a larger power law relationship of 0.95 ( ± 0.0061), suggesting a more equal distribution of large and connected clusters (Fig. 3b). Of note, the HCQ (0.78 ± 0.018) and TMZ (0.80 ± 0.005) networks presented with the lowest gradients (Fig. 3c), indicative of smaller area and perimeter values due to the large amount of fragmentation.

### Chemotherapeutic resistance is associated with complex 1 linked OXPHOS and is diminished through coordinated autophagy modulation

**Figure 4:**
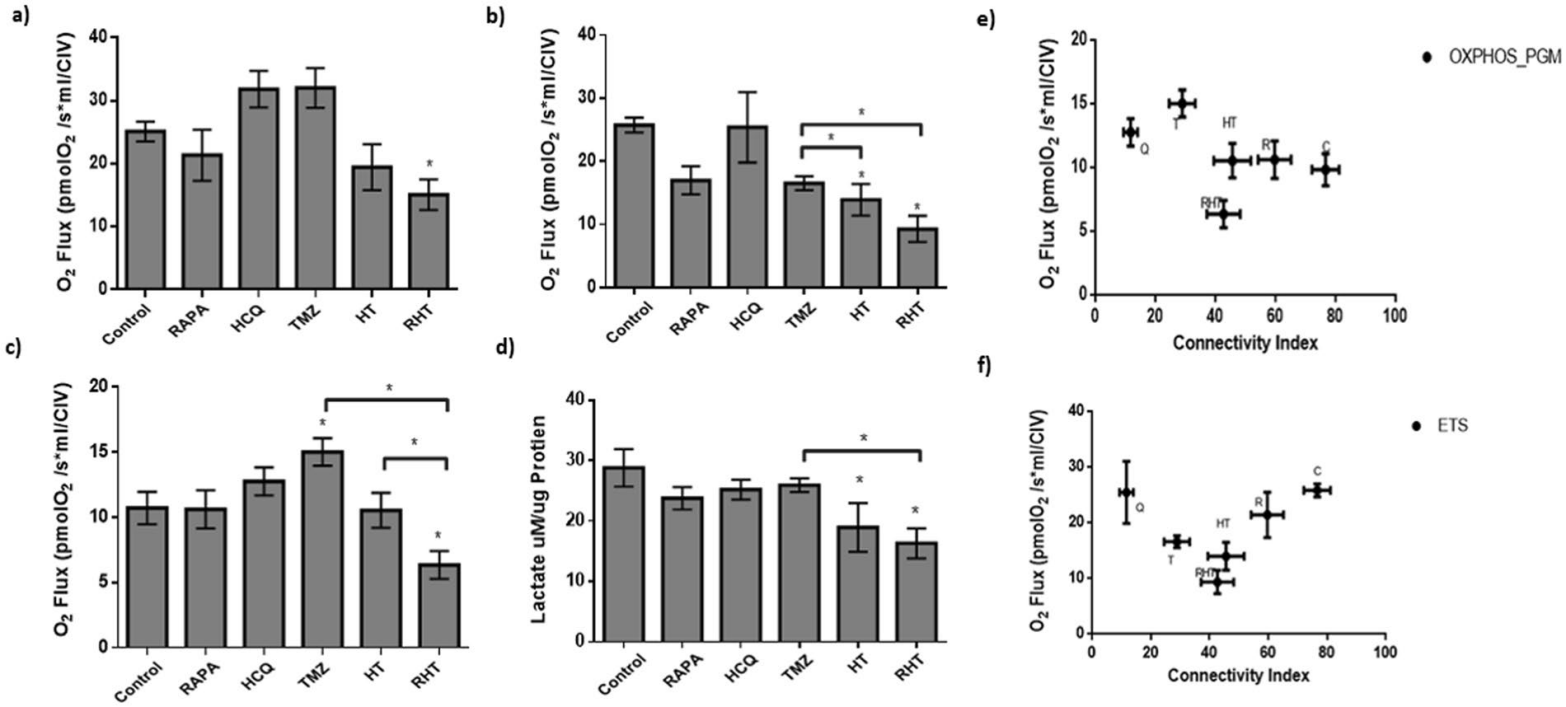
Chemotherapeutic resistance is mediated by complex 1 linked OXPHOS and is diminished through coordinated autophagy modulation. (**a**) Routine (**b**) Maximal ETS response and (**c**) OXPHOS through Complex I associated O_2_ Flux values and (**d**) concentration of lactate in growth medium, (**e**) correlation of O_2_ flux with Connectivity Index for all groups of interest for (**e**) OXPHOS through Complex I and (**f**) ETS Capacity for Control, 50 nM Rapamycin (Rapa), 50 µM Hydroxychloroquine (HCQ), 250 µM Temozolomide (TMZ), HCQ (50 µM) + TMZ (250 µM) (HT) and Rapa (50 nM) + HCQ (50 µM) + TMZ (250 µM) (RHT) treatment groups. All error bars, ±SEM. *p < 0.05, n = 3.

A bioenergetic profile comprising of key metabolic states was generated following a SUIT protocol that was applied to permeabilised U-118MG cells and assessed through high resolution respirometry. Routine respiration and ETS capacity was not altered significantly by autophagy induction (Rapamycin, 50 nM), autophagy inhibition (HCQ, 50 µM) or treatment with chemotherapy (TMZ, 250 µM) compared to control cells (Fig. 4a,b). However, HT treatment, as well as Rapamycin pre-treatment in combination with HT (RHT) decreased the maximal ETS capacity in comparison to both the control and TMZ treatment groups (Fig. 4b). Complex I linked OXPHOS was unperturbed by treatment with Rapamycin and HCQ (Fig. 4c), yet it was significantly enhanced following 24 hours incubation with TMZ and it decreased back to the same level as the control following pre-treatment with HCQ (Fig. 4c). Following the RHT regimen resulted in a significant decrease in complex I linked OXPHOS compared to Control, TMZ and HT treatment groups (Fig. 4c). From this, it can be deduced that OXPHOS through complex 1 is necessary to maintain mitochondrial respiration under chemotherapeutic conditions and that this response can be diminished through coordinated autophagy modulation.

### Autophagy modulation in combination with TMZ decreased lactate production

In order to assess anaerobic glycolysis, the concentration of lactate produced per microgram protein was determined in culture media. Under control conditions, lactate production was measured at 30 µM/µg (±3.11 µM/µg), which remained unaltered after upregulation of autophagy with Rapamycin (50 nM), inhibition with HCQ (50 µM) and treatment with TMZ (250 µM) for 24 hours (Fig. 4d). Pre-treatment with HCQ (HT) and Rapamycin (RHT) decreased lactate production significantly to 18.95 µM/µg (±4.03 µM/µg vs Control p < 0.05) and 16.31 µM/µg (±2.47 µM/µg vs Control, p < 0.05) (Fig. 4d). Given that OXPHOS was also decreased in these groups, decreased autophagic activity seemed to impair both aerobic and anaerobic glycolytic pathways.

### Mitochondrial Form and Function

**Figure 5:**
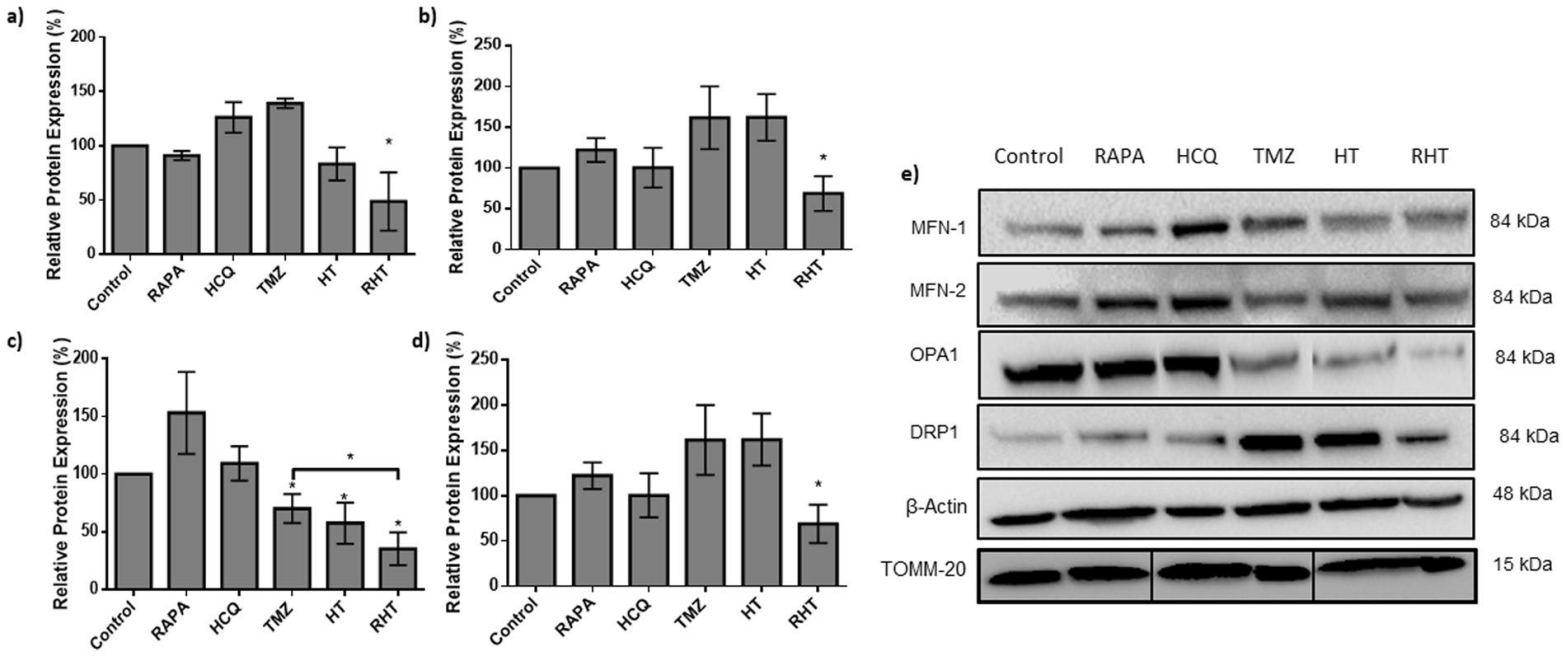
Decreased expression of MFN1, MFN2, OPA1 and DRP1 is caused by coordinated autophagy modulation. Relative protein expression levels of (**a**) MFN1, (**b**) MFN2, (**c**) OPA1 and (**d**) DRP1 in U-118MG cells. (**e**) Representative Immunoblot of relative Mitofusin 1 (MFN1) protein levels for Control, 50 nM Rapamycin (Rapa), 50 µM Hydroxychloroquine (HCQ), 250 µM Temozolomide (TMZ), HCQ (50uM) + TMZ (250 µM) (HTMZ) and Rapa (50 nM) + HCQ (50 µM) + TMZ (250 µM) (RHT) treatment groups. N = 3. *p < 0.05.

Correlating O_2_ flux with Connectivity Index data indicates that although the HCQ and TMZ groups presented with the least amount of connectivity, their OXPHOS (Fig. 5a) associated respiration was above that of healthy control cells. Importantly, the two intermediately connected groups HT and RHT produced the least effective ETS response (Fig. 5b). These data suggest that attributing bioenergetic efficiency to morphological changes in isolation of respiratory analysis can be misleading and may mask the true cellular stress response.

### Decreased expression of MFN1, MFN2, OPA1 and DRP1 is caused by coordinated autophagy modulation

**Figure 6:**
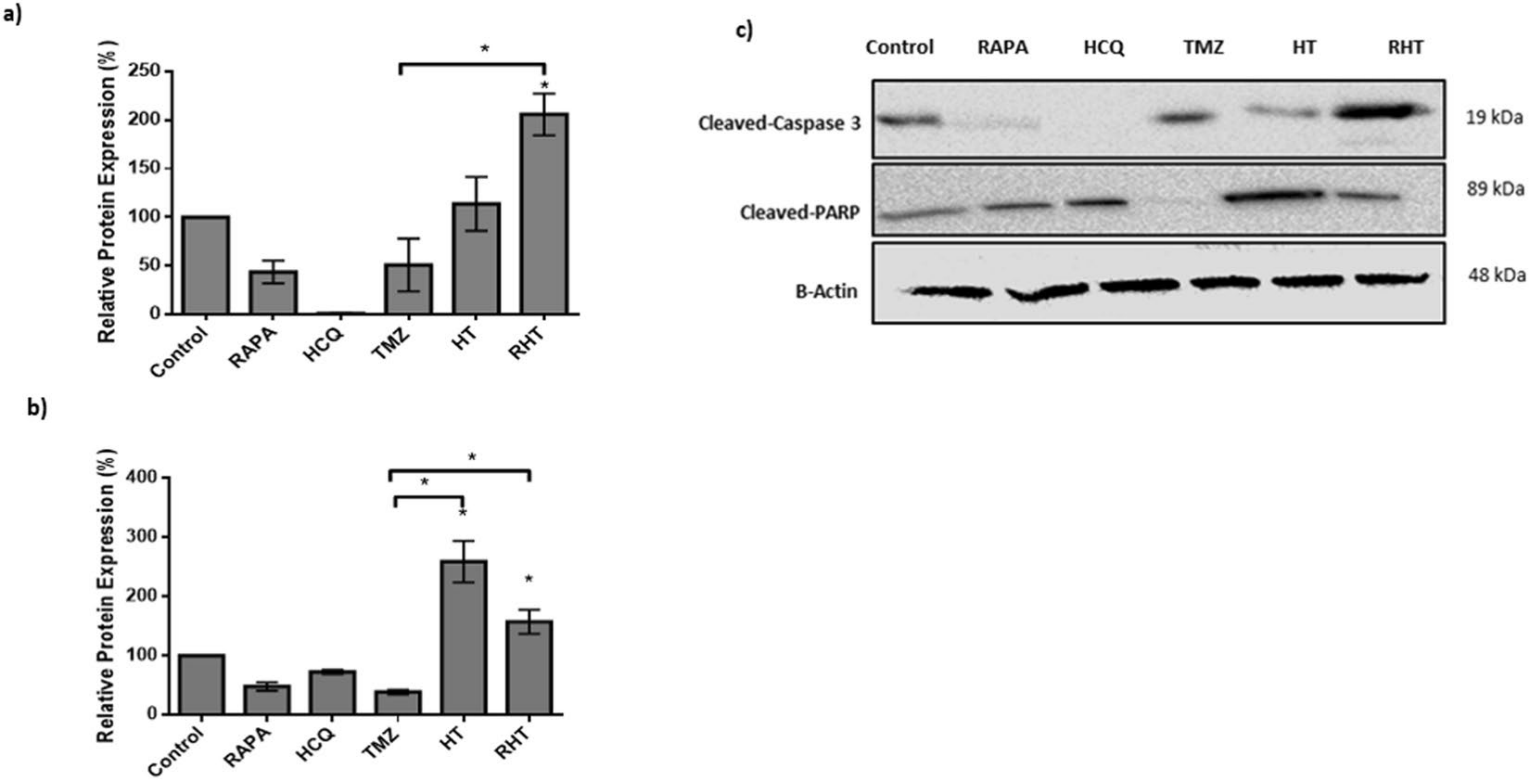
Cell death induction is only apparent in the RHT treatment group of U-118MG cells. (**a**) Relative protein expression levels of cleaved PARP. (**b**) Relative protein expression levels of cleaved Caspase 3. (**c**) Representative Immunoblot of relative cleaved PARP and cleaved caspase-3 expression levels for Control, 50 nM Rapamycin (Rapa), 50 µM Hydroxychloroquine (HCQ), 250 µM Temozolomide (TMZ), HCQ (50 µM) + TMZ (250 µM) (HT) and Rapa (50 nM) + HCQ (50 µM) + TMZ (250 µM) (RHT) treatment groups. N = 3, *p < 0.05 vs corresponding Baf negative group. N = 3. *p < 0.05, ***p < 0.001.

In order to determine the molecular mechanisms responsible for the changes in dynamics and morphology observed in sections 3.2 and 3.3, the expression of key fusion and fission proteins was assessed through Western Blot analyses. These include the fusion proteins MFN1, MFN2 and OPA-1 as well as the fission protein DRP1. MFN1 protein levels remained unaltered following Rapamycin, HCQ and TMZ treatment (Fig. 6a,e). Pre-treatment with HCQ for 6 hours also had no effect on MFN1 expression (Fig. 6a,e). However, 6 hour pre-treatment with Rapamycin followed by HCQ and TMZ decreased MFN1 expression significantly compared to the control (Fig. 6a,e).

MFN2 expression levels followed much the same trend as MFN1, with unaltered signal intensities observed for Rapamycin, HCQ, TMZ and HT treatment groups (Fig. 6b,e). A moderate, yet significant decrease in MFN2 expression was observed after RHT treatment compared to control cells (Fig. 6b). Autophagy induction through Rapamycin did not alter OPA-1 expression levels, nor did inhibition through CQ (Fig. 6c). However, 24 hours of TMZ (250 µM) treatment decreased OPA-1 protein levels significantly compared to Control, with the same effect observed for the HT (HCQ pre-treatment) group (Fig. 6c). Upregulating autophagy for 6 hours (Rapamycin, 50 nM) prior to incubation with HCQ (50 µM) and treatment with TMZ (250 µM) decreased OPA-1 expression considerably compared to both the control and TMZ groups (Fig. 6c), suggesting that both inner and outer membrane fusion was impaired.

Induction of autophagy for 6 hours with Rapamycin did not alter DRP1 protein expression, nor did 6 hours of autophagy inhibition with HCQ (Fig. 6d). The same was observed following 24 hours incubation with TMZ (250 µM) and 6 hours co-incubation with HCQ (Fig. 6d). However, RHT treatment decreased DRP1 expression significantly compared to the control (Fig. 6d,e), which indicates that regulation of both key fission and fusion proteins was impaired in the RHT group which displayed lowest mitochondrial respiratory activity.

### Apoptotic Cell death induction is only apparent in the RHT treatment group

**Figure 7:**
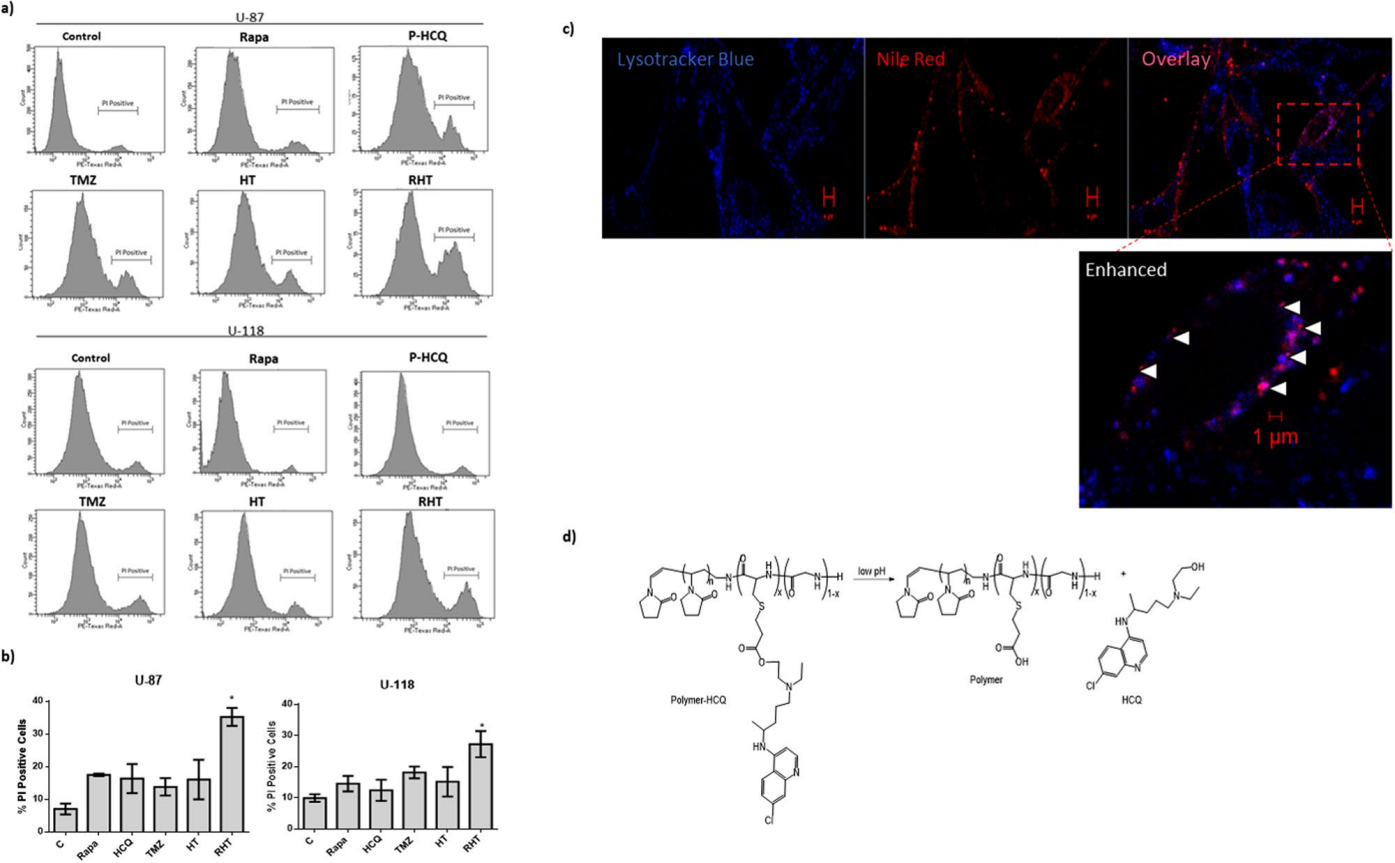
Cell death is only apparent for RHT treatment in both U-87 and U-118 MG cells. Propidium Iodide (PI) uptake quantification for both cell lines is shown in (**a**) and (**b**) treated with 50 nM Rapamycin (Rapa), 50 µM Hydroxychloroquine-polymer (P-HCQ), 250 µM Temozolomide (TMZ), 50 µM (P-HCQ) + TMZ (250 µM) (HT) and Rapa (50 nM) + P-HCQ (50 µM) + TMZ (250 µM) (RHT). P-HCQ treated U-118 MG cells stained with Lysotrakcer Blue and Nile Red (Scale Bar 5 µM) is shown in (**c**) as well as overlay between Lysotracker Blue and Nile red indicated in the enhanced image, confirming that the HCQ-polymer reached lysosomes (Scale Bar 1 µM). (**d**) Schematic representation of the release of the (acid labile) β-thiopropionate linked hydroxychloroquine drug from the poly(N-vinylpyrrolidone-block-(cysteine-co-glycine)) [PVP-b-rPp] (Pp = polypeptide). *p < 0.05, n = 3. All error bars, ± SEM.

Cleavage of caspase 3 is a result of apoptosis induction through the intrinsic pathway and results in cleavage of the DNA repair enzyme PARP. Cleavage of PARP renders it incapable to counter act DNA damage, the accumulation of which results in cell death onset. Relatively unaltered cleaved caspase-3 expression was observed for Rapamycin and HCQ treatment groups (Fig. 7a,c). Cleaved caspase-3 protein levels were diminished following 6 hours incubation HCQ (Fig. 7a,c). HCQ pre-treatment (50 µM, 6 hours) followed by TMZ (250 µM, 24 hours) also decreased cleaved caspase 3 protein levels. However, inducing autophagy with Rapamycin (50 nM) 6 hours prior to incubation with HCQ (50 µM, 6 hours) and TMZ (250 µM, 24 hours) resulted in significantly increased cleaved caspase-3 protein levels (Fig. 7a,c). Cleaved-PARP protein levels were unaltered when upregulating autophagy with Rapamycin (50 nM) as well as after inhibition of autophagy with HCQ (Fig. 7b,c). 24 hours incubation with TMZ also did not alter cleaved-PARP expression. However, co-incubation of HCQ with TMZ for 6 hours significantly enhanced cleaved-PARP expression (Fig. 7b,c). Pre-treatment with Rapamycin, followed by HCQ and TMZ treatment also increased cleaved-PARP protein levels significantly (Fig. 7b,c). Therefore, only the RHT treatment group displayed the enhanced protein levels of both cleaved PARP and cleaved caspase 3 necessary to indicate apopotosis onset.

To better quantify cell death onset and rule out cell line specificity, both U-87 and U-118MG cells were stained with propidium iodide (PI) and its uptake was quanitified via flow cytometery. PI is a DNA-intercalating agent that is not inherently cell-permeable and can therefore be used to measure viability as dying cells have compromised membrane integrity. Furthermore, to ensure that the decreased viability observed in the HCQ, HT and RHT treatment groups were not due to off target effects of HCQ, a novel polymer was designed to pH dependently deliver HCQ to lysosomes (Fig. 7c,d). Polypeptide-based pH responsive polymers are well reported as nanocarriers for bioactive molecules^23,30^. Hence we prepared a new nanocarrier, where hydroxychloroquine is covalently attached via an acid-labile β-thiopropionate bond. The acid-labile β-thiopropionate linker has been employed for nonpolypeptide (conventional polymers) based pH responsive polymers and can release drugs or bioactive molecules near endosomal pH (6 to 5)^31,32^.

PI viability assays indicate that the HCQ-polymer did not significantly enhance cell death when treated in isolation (50 µM for 6 h), although when used in combination with rapamycin and TMZ a marked increase in cell death for the RHT treatment group was observed (Figs 6 and 7). These results correlate well with the decreased reductive capacity seen in the initial WST-1 assays (Fig. 1a) and the increased presence of apoptotic proteins (Fig. 6a–c).

**Figure 8:**
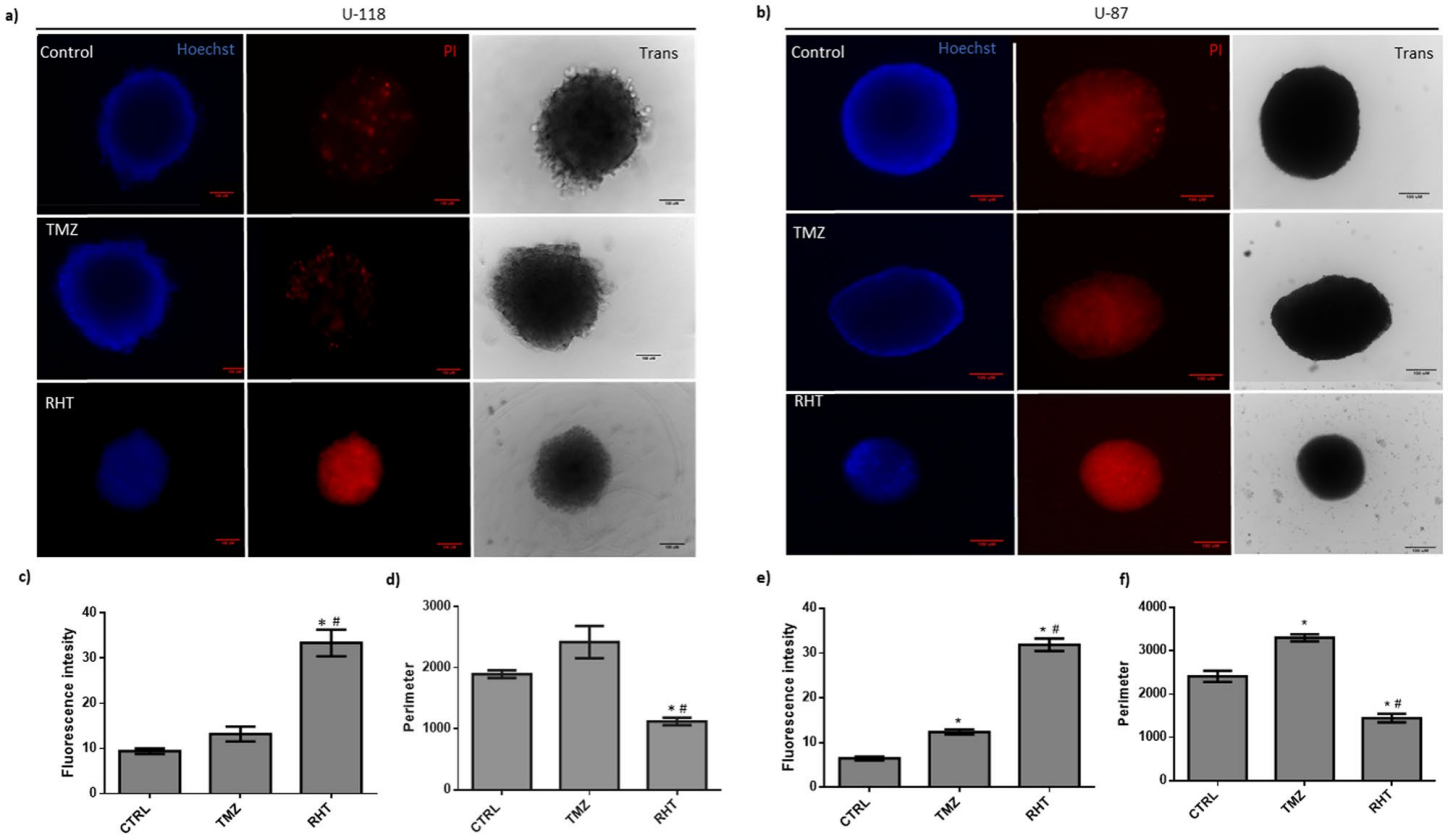
Assessment of 3D spheroid viability and topology changes under modified TMZ and RHT treatment. U-118MG spheroids stained with Hoescht (blue signal) and propidium iodide (PI) are shown in (**a**) and (**b**). PI Fluorescence intensity for (**c**) U-118MG spheroids and (**e**) U-87 spheroids and the outer perimeter of spheroids are shown in (**d**) for U-118MG spheroids and (**f**) for U-87 spheroids. Treatments consisted of 1 mM TMZ over 72 hours or 500 nM Rapamycin for 24 hours followed by 500 µM P-HCQ for 24 hours followed by 1 mM TMZ for 72 hours (RHT). *p < 0.05 vs Control, ^#^p < 0.05 vs TMZ, n = 8. Scale Bar 100 µM.

Lastly, to assess the clinical translatability of these findings, the RHT treatment regimen was compared to chemotherapeutic treatment in a 3D tumour spheroid model. It has been established previously that cancer spheroids display considerably more resistance to chemotherapy than monolayer cultures^33^. Indeed, we found that staining U-87 and U-118 spheroids with PI using the same rapamycin, HCQ and TMZ concentrations as for their 2D counterparts revealed no significant increase in signal intensity or sphere size following the RHT regime. However extending the treatment duration for both rapamycin and HCQ to 24 hours (500 nM and 500uM respectively) and TMZ treatment to 72 hours (1 mM) revealed enhanced spheroid cell death and decreased size (Fig. 8).

## Discussion

The present study attempted to elucidate whether autophagy modulation would impair GBM metabolism to the extent of initiating cell death. Combining autophagy induction with subsequent inhibition was hypothesised to debilitate glioma metabolism most prominently in terms of ETS and OXPHOS capacities and mitochondrial fission and fusion functionality. We have shown that chemotherapeutic resistance can be overcome through carefully coordinated autophagy modulation consisting of both induction and inhibition periods. Importantly, robust changes in autophagic degradation activity resulted in the distinct alteration of mitochondrial OXPHOS capacity.

Enhanced degradation through autophagy and complex I linked OXPHOS was observed in response to TMZ treatment, indicating that autophagy and mitochondrial respiration is essential for GBM survival under conditions of cytotoxic stress. Given that protein extraction occurred at a single time point, regardless of the rate of autophagic degradation, the decrease in initial LC3-II expression following TMZ treatment is representative of an already enhanced flux and not necessarily a decrease in autophagosome production^34^. This is supported by the significant increase in LC3-II expression caused by Baf treatment, indicative of an increase in autophagic flux. The robust response in LC3-II accumulation following HT treatment strengthens the notion that TMZ enhanced autophagic flux (Fig. 1). It has previously been hypothesize that an increased degradative activity enhances the availability of autophagy derived substrates, serving as an adaptive response to meet cellular metabolic demands^7,35^. Therefore, in the present study, lysosomal deacidification occurred prior to chemotherapy mediated autophagy induction, rendering autophagolysosomes with impaired degradative ability. In addition, our results revealed that enhanced autophagosomal flux induced by Rapamycin exacerbates this effect. By increasing the autophagosomal pool size prior to lysosomal deacidification (RHT) it is possible that autophagosome production was enhanced beyond maximal capacity, rendering the TMZ response ineffective (Figs 1 and 4).

To elucidate whether mitochondrial bioenergetics were indeed affected by autophagy modulation, the mitochondrial phenotype was assessed in terms of its fission and fusion dynamics. The regulation of both fission and fusion events have recently been implicated in metabolic efficiency^17,18^. In our study, a gradual loss of signal intensity was observed over time for cells under control conditions indicative of a highly motile network (Fig. 2). Linear regression through these slopes indicate significantly slower singal dissipation in all treatment groups, which was further exacerbated by ATG5 siRNA knockdown (Fig. 2, Supplementary Fig. 2) in both cell lines tested. Importantly, unaltered mitofusin protein expression (MFN1 and MFN2) was observed for treatment groups that displayed the least amount of connectivity, indicating that outer membrane fusion was not impaired (Fig. 5a,b,e), in the U-118 MG cell line at least. Similarly, DRP1 protein levels were not enhanced in these groups (HCQ and TMZ), indicating that enhanced fission was not solely responsible for enhanced fragmentation (Fig. 5d,e). However, decreased OPA1 protein levels were observed in fragmented and intermediately connected networks, indicating that diminished inner membrane fusion resulted in incomplete fusion (Fig. 5a,e). Therefore, loss of OPA1 protein levels proved a better indicator of decreased network connectivity than a decrease in DRP1 protein levels, suggesting that decreased connectivity was more likely attributable to decreased inner membrane fusion and not increased fission. In this regard, RHT treatment caused a significant decrease in both OPA1 and DRP1 expression (Fig. 5), indicating that decreased fission regulation altered morphological distribution, given that larger and more individual clusters were observed in RHT treated cells compared to TMZ and HT (Fig. 3d).

Extensive investigation has recently been conducted on the involvement of fission and fusion proteins in metabolic sensing and mitochondrial efficiency. Mishra *et al*. (2014) have reported that mouse embryonic fibroblast (MEF) cells devoid of MFN1 did not decrease their OXPHOS capacity^17^. However, OPA1 cleavage has been shown to be affected by altered OXPHOS requirements, suggesting that inner membrane fusion is more critical to electron transport chain efficiency than outer membrane fusion^17^. Recent observations revealed that the fission regulator, DRP1, is of equal importance, as silencing DRP1 impaired growth, decreased oxygen consumption and induced apoptosis in brain tumour initiating cells (BTICs)^18^. Furthermore, AMPK activation was shown in BTICs under these conditions, indicating that impaired DRP1 expression can lead to metabolic stress in cancer cells^18^. Functionally, fragmented mitochondrial networks have been observed in cell types with defective OXPHOS^36–38^. However, OXPHOS impaired primary human fibroblasts also display no apparent morphological changes^38–40^. Contrary to earlier studies, increased fragmentation was not associated with decreased respiratory capacity^41^. In fact, we have shown that OXPHOS capacity remained unaltered in HCQ treated cells that displayed the largest amount of fragmentation (Fig. 4). Surprisingly, our data indicate that an increase in OXPHOS, as observed in TMZ treated cells, can indeed be associated with decreased mitochondrial connectivity (Figs 3 and 4). Therefore, it is possible that mitochondrial fragmentation served as an early adaptive mechanism to chemotherapy in glioma cells and highlights the need for further investigation into mitochondrial spatiotemporal changes in this scenario.

MFN1 and MFN2 expression remained unaltered in OXPHOS capable treatment groups, although RHT treated cells presented with decreased MFN1 and MFN2 expression (Fig. 5). In accordance with recent literature, decreased OPA1 expression was associated with impaired ETS capacity^17^. DRP1 expression also remained unaltered for the Rapamycin, HCQ and HT treatment groups (Fig. 5). Although network fragmentation was observed following HCQ, TMZ and HT treatment, these groups displayed sufficient OXPHOS capacity, strengthening the case for DRP1 as an important metabolic regulator (Figs. 4 and 5). Consequently, the decreased OXPHOS and ETC capacities observed in the RHT treated cells supports this notion. Furthermore, impaired autophagic activity was associated with decreased lactate production (Fig. 5). Importantly, U-118MG cells that displayed diminished OXPHOS and glycolysis were also found to have enhanced cleaved caspase 3 and cleaved PARP protein levels. This indicates that cells were metabolically compromised to the extent of inducing apoptotic cell death. In this regard, it has been shown that following MOMP, caspase 3 is capable of cleaving the p75 subunit of complex I, decreasing its respiratory efficiency in HeLa cells^42^. Our results support these findings, as an increase in caspase3 and decrease in complex I linked OXPHOS was observed for the RHT treatment group. Increased PI uptake in both U-87 and U-118 cell lines and 3D spheroid models under RHT treatment further confirmed increased cell death sensitization through coordinated autophagy modulation (Fig. 8).

Clinical trials have been conducted to assess the combination of HCQ with TMZ with only stable disease state achieved^9^. However, a more potent inhibitor of autophagy, metformin, has shown great promise in reducing glioblastoma chemoresistance both *in vitro* and *in viuvo*, suggesting that upstream inhibition of autophagy has greater anti-cancer properties than late stage inhibitors such as HCQ^43^. In this regard, we have shown that by attenuating the release mechanism of HCQ, late stage autophagy inhibiton can still impair tumour viability in combination with TMZ. Furthermore, given that the flux dependent nature of autophagy is rarely taken into account when determining modulator dosages in cancer patients, the possibility exists that the difference in tumour autophagic flux and healthy tissue offsets the efficiency of dose escalation studies. This is evident in the recent clinical trial combining temsirolimus (TEM), a rapamycin analog, and HCQ, where only stable disease state was reached in patients with advanced solid tumours^44^.

In the current study, we have shown that disruption of the autophagic system is possible through coordinated modulation with drug concentrations based on relative flux assessments in addition to toxicitiy assays. We have demonstrated that such modulation was effective in impairing the ability of GBM cells to evade chemotherapy-induced cell death. Impaired autophagic degradation resulted in decreased mitochondrial respiration, with OXPHOS being impaired to a greater extent in intermediately fragmented rather than completely fragmented networks. These findings may be of clinical importance and highlights the dependency of GBM survival on the interaction between the autophagic system and mitochondrial bioenergetics. Future work is required to enhance clinical translation of coordinated autophagy modulation as a clinical therapeutic intervention for GBM.

## Electronic supplementary material

Supplementary data
