# Supplementary material for: Coordinated autophagy modulation overcomes glioblastoma chemoresistance through disruption of mitochondrial bioenergetics
Source: Sci Rep. 2018 Jul 9;8:10348. doi: 10.1038/s41598-018-28590-9 (PMC6037778; doi:10.1038/s41598-018-28590-9)
Supplement: Supplementary file 1 — Supplementary data [file 41598_2018_28590_MOESM1_ESM.docx]

**Coordinated autophagy modulation overcomes glioblastoma chemoresistance through disruption of mitochondrial bioenergetics**

**Supplementary Information**

Jurgen Kriel^a^, Kristian Müller-Nedebock^b^, Gerald Maarman^c^, Siyasanga Mbizana^d^, Edward Ojuka^c^, Bert Klumperman^d^, and Ben Loos^a^

^a^ Department of Physiological Sciences, Faculty of Science, University of Stellenbosch, Stellenbosch, 7600, South Africa.

^b^ Department of Physics, Faculty of Science, University of Stellenbosch, Stellenbosch, 7600, South Africa

^c^ Division of Exercise Science and Sports Medicine Institute (ESSM), Department of Human Biology, University of Cape Town, 7700, South Africa

^d^ Department of Polymer Science, Faculty of Science, University of Stellenbosch, Stellenbosch, 7600, South Africa.

Corresponding Author:

**Ben Loos:**

Tel: +27 21 808 9196

Fax: +27 21 808 3145

E-mail: [bloos@sun.ac.za](mailto:bloos@sun.ac.za)

**
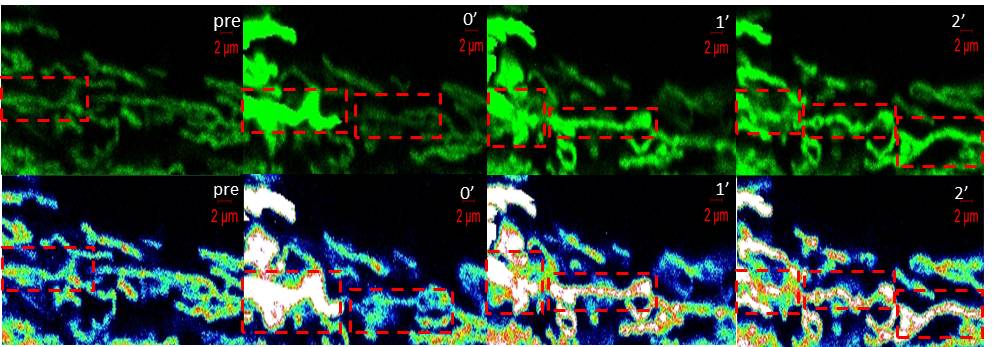
**

**Supplementary Figure 1**. **Photoactivation assay.** Representative images of mito-PA-GFP transfer with corresponding look up table (LUT) intensities as a result of fusion events. Initial region of interest (ROI) signal intensity pre-activation was 90,41 (Pre). Following photoactivation, an increase in signal intensity was observed for the ROI (207,35 at time of activation/0 minutes), with the neighbouring region remaining 80.00. 1 min post-activation, spread of signal intensity was observed, with intensity values of 158,00 for the initial activated region and 128,92 for the neighbouring region. 2 min post activation, further transfer of signal intensity was observed, with intensity values of 133,59, 108,623 and 119,49 for the three ROIs respectively


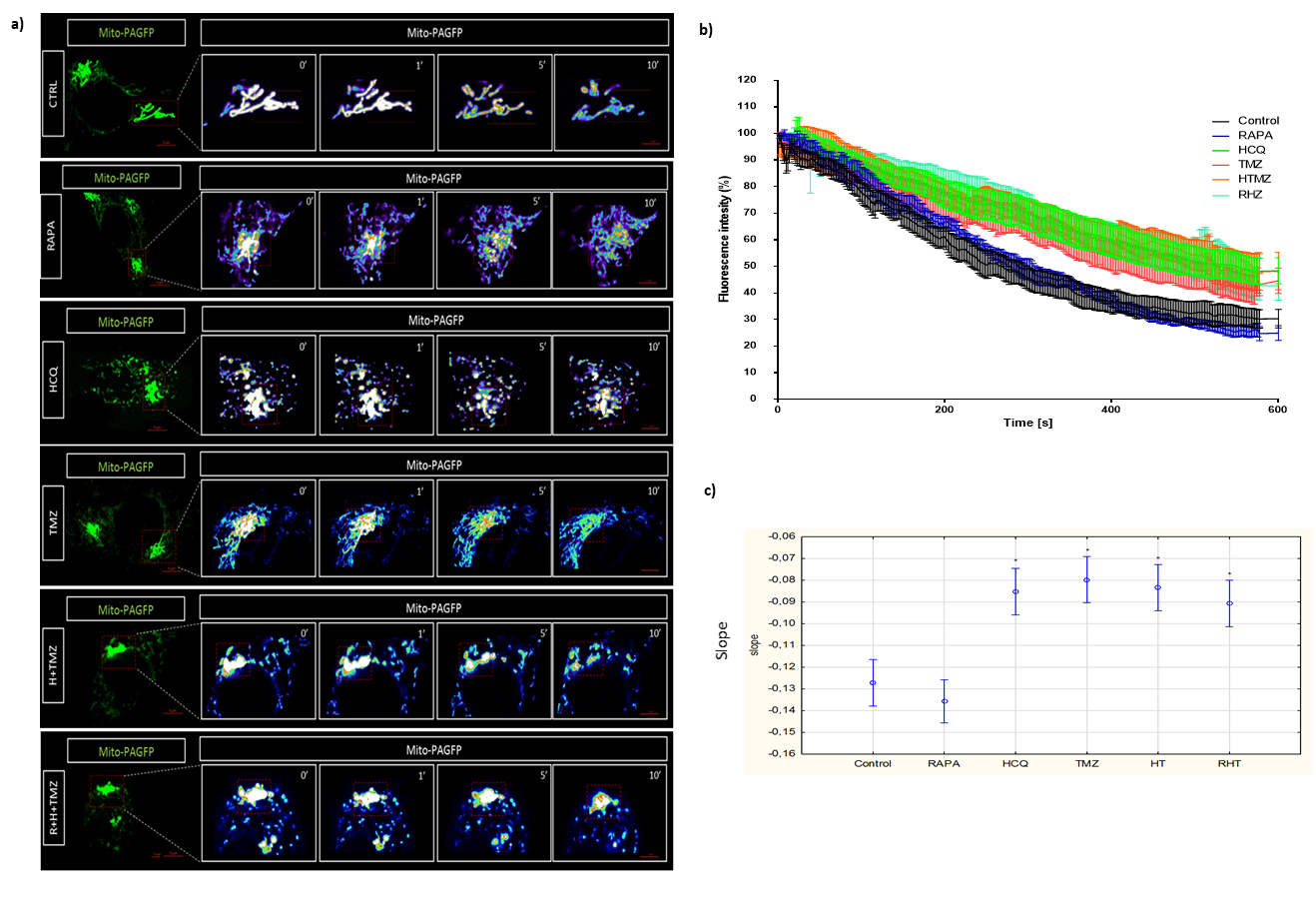


**Supplementary Figure 2. Coordinated autophagy modulation disrupts mitochondrial fission and fusion dynamics.** a) Representative image of mito-PA-GFP transfected cells (60X magnification, scalebar 5μM) with the activated region outlined in red enhanced to display LUT intensities at 0, 1, 5 and 10 minute intervals post activation. b) Decrease of mito-PA-GFP signal over time, represented as percentage of the initial signal intensity. c) Slope comparison of linear regressions through signal decay curves. All error bars, ± SEM.*p <0.05 vs control, n=6.


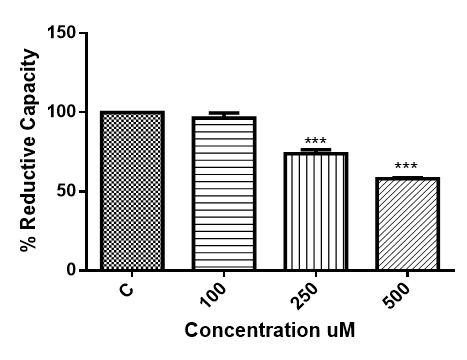


**Supplementary Figure 3. Prolonged incubation with TMZ**. WST 1 viability assays of U-118MG cells treated with 100, 250 and 500 μM of Temozolomide (TMZ) for 48 hours. n=3, **p < 0.01 vs Control, ***p<0.001 vs Control.


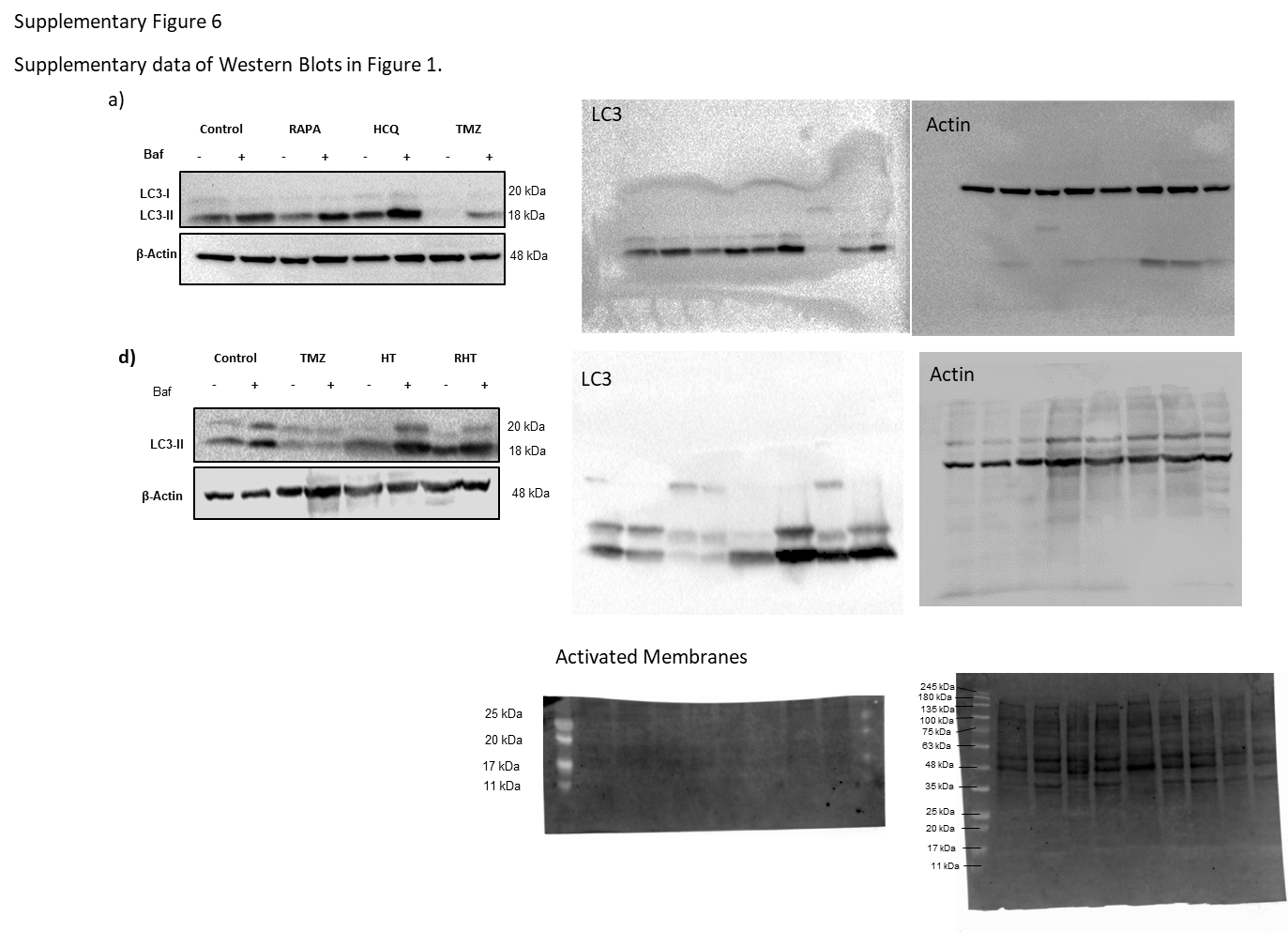


**Supplementary Figure 5.** Supplementary data of Western Blots in Figure 1**.** Original membranes of each cropped area are provided as well as the activated membranes with ladder size indications.


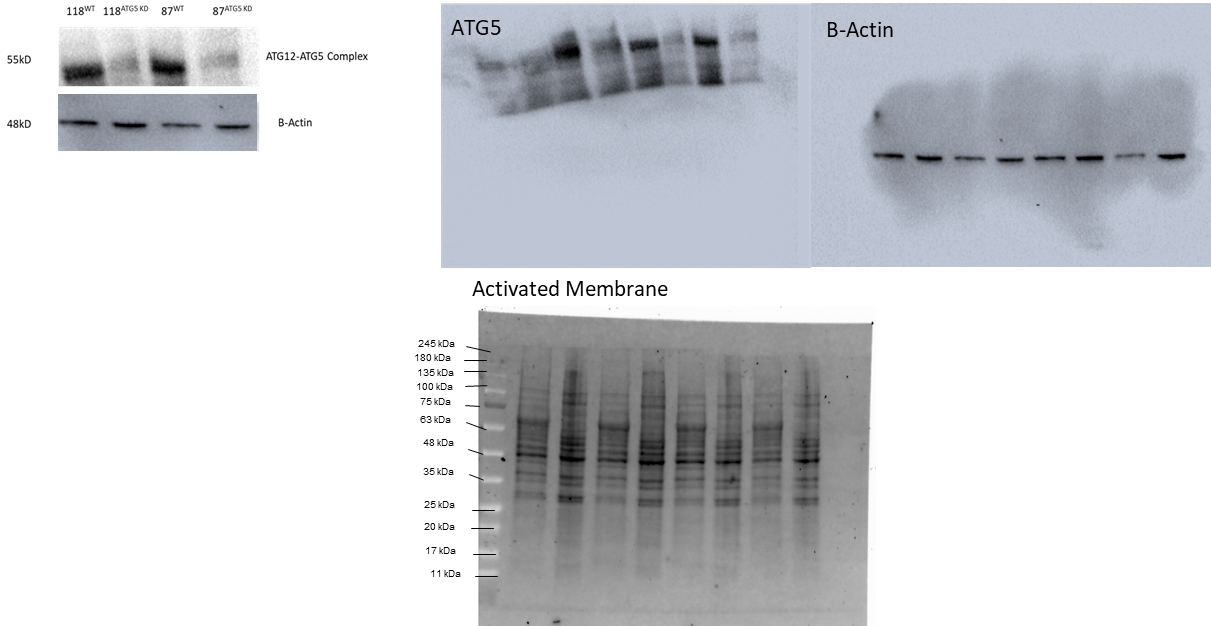


**Supplementary Figure 6.** Supplementary data of Western Blots in Figure 2**.** Original membranes of each cropped area are provided as well as the activated membranes with ladder size indications

**
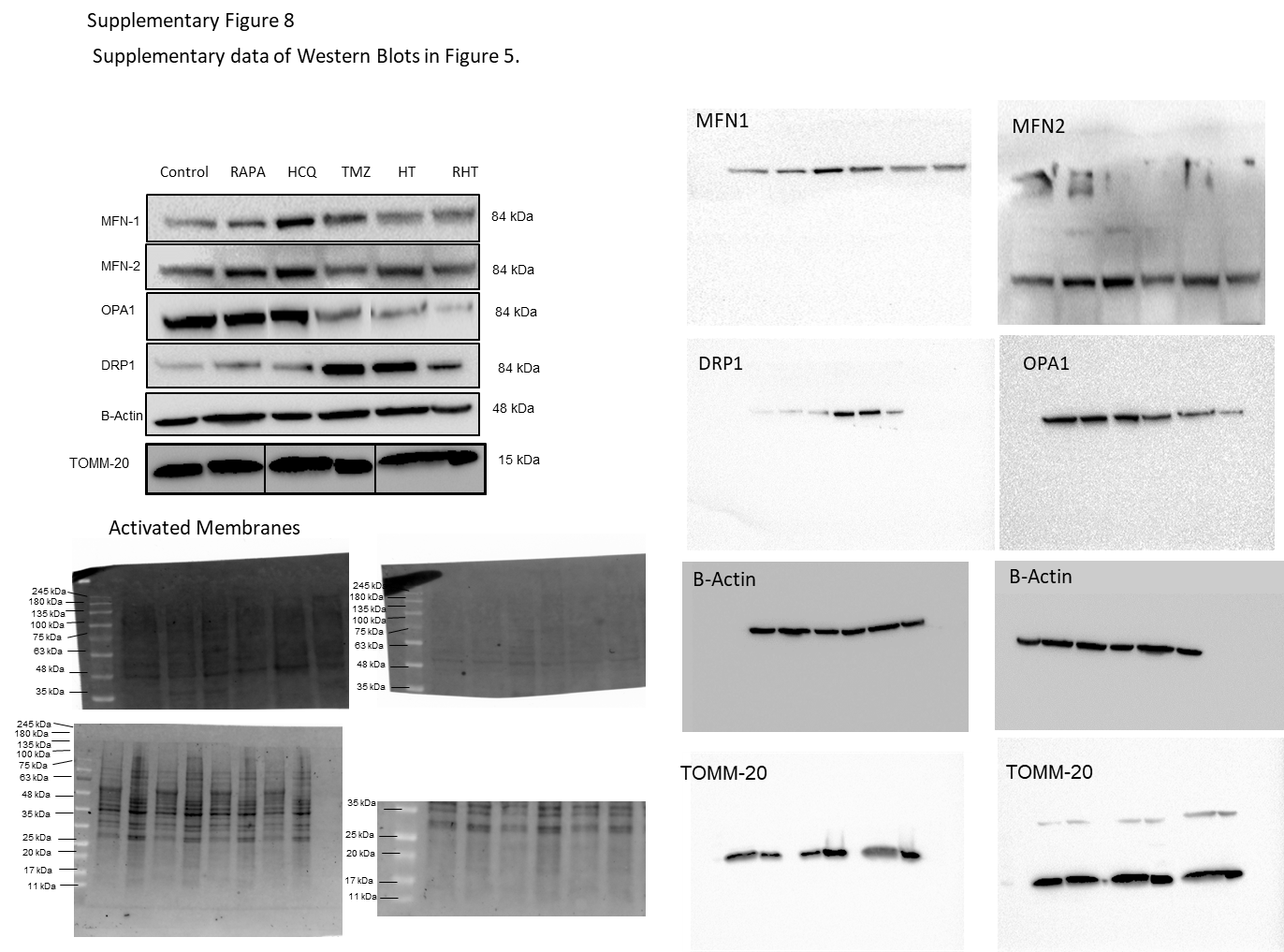
**

**Supplementary Figure 7.** Supplementary data of Western Blots in Figure 5**.** Original membranes of each cropped area are provided as well as the activated membranes with ladder size indications.


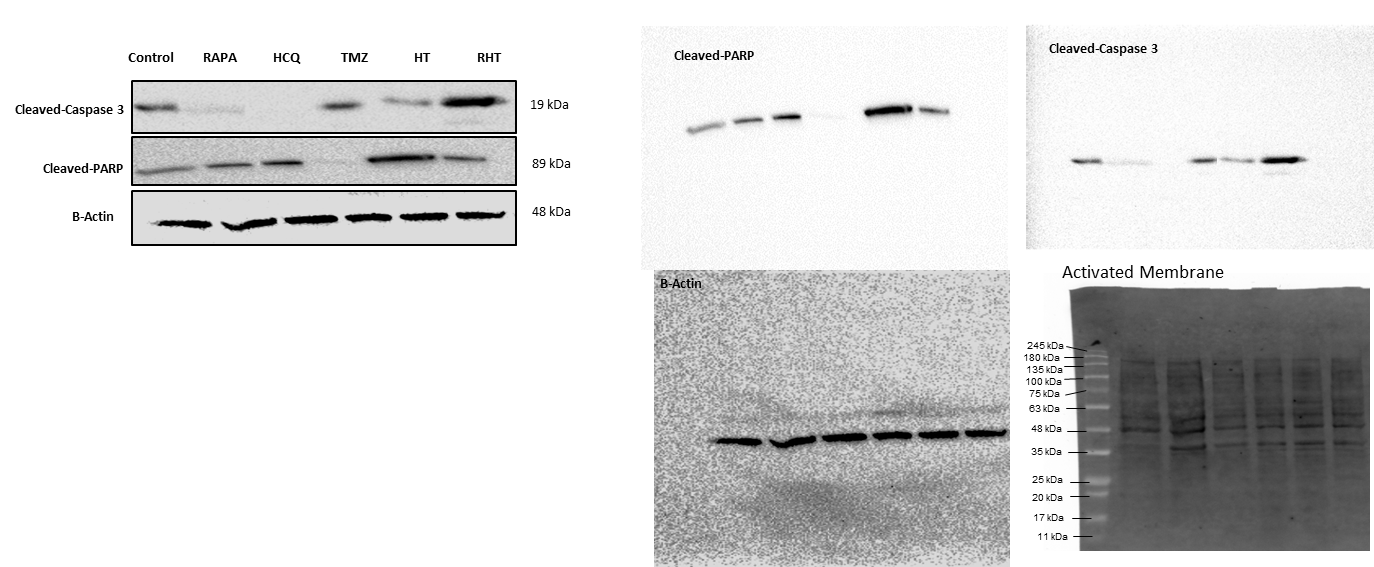


**Supplementary Figure 8.** Supplementary data of Western Blots in Figure 6**.** Original membranes of each cropped area are provided as well as the activated membranes with ladder size indications
